# Effect of resolvin D5 on T cell differentiation and osteoclastogenesis analyzed by lipid mediator profiling in the experimental arthritis

**DOI:** 10.1038/s41598-021-96530-1

**Published:** 2021-08-27

**Authors:** Hirotaka Yamada, Jun Saegusa, Sho Sendo, Yo Ueda, Takaichi Okano, Masakazu Shinohara, Akio Morinobu

**Affiliations:** 1 Department of Rheumatology and Clinical Immunology, Kobe University Graduate School of Medicine, Kobe, Japan; 2 Department of Clinical Laboratory, Kobe University Hospital, Kobe, Japan; 3 Division of Epidemiology, Kobe University Graduate School of Medicine, Kobe, Japan; 4 The Integrated Center for Mass Spectrometry, Kobe University Graduate School of Medicine, Kobe, Japan

**Keywords:** Rheumatology, Immunological disorders, Inflammation

## Abstract

Resolvins, are specialized pro-resolving mediators (SPMs) derived from n-3 polyunsaturated fatty acids. They contribute actively to the resolution of inflammation, but little is known concerning their role in chronic inflammation, such as in rheumatoid arthritis (RA). Here, we performed lipid mediator (LM) profiling in tissues from the paws of SKG arthritic mice using lipid chromatography (LC)/mass spectrometry (MS)/MS-based LM metabololipidomics. We found elevated levels of SPMs including resolvin D5 (RvD5) in these tissues. Moreover, RvD5 levels were significantly correlated with arthritis disease activity. From experiments to assess the role of RvD5 in the pathology of RA, we concluded that RvD5 suppressed Th17 cell differentiation and facilitated regulatory T cell differentiation, as well as inhibiting CD4^+^ T cell proliferation. Furthermore, RvD5 attenuated osteoclast differentiation and interfered with osteoclastogenesis. Targeting the resolution of inflammation could be promising as a novel treatment for RA.

## Introduction

Over the last two decades, it has become increasingly clear that resolution of acute inflammation is not a passive process, but requires active modulation^1,2^. This is tightly regulated by families of novel potent bioactive LMs, which have been termed “specialized pro-resolving mediators” (SPMs)^3,4^. These are synthesized from inflammatory exudate and stimulate the resolution phase via enhancement of neutrophil apoptosis, macrophage infiltration, M2-type polarization and efferocytosis to return to the baseline homeostatic state^5^. When acute inflammation fails to resolve appropriately and leads to chronic inflammation, it is thought that dysregulation of these SPMs may be involved in chronic disorders such as rheumatoid arthritis (RA)^4^. RA is a chronic inflammatory disease characterized by synovial inflammation and hyperplasia, autoantibody production, and bone destruction^6^. These features are caused by abnormalities in adaptive and innate immune processes^7^. Interactions between leukocytes, synovial fibroblasts and OCs drive the chronic phase in the pathogenesis of RA^8^.

The effectiveness of n-3 polyunsaturated fatty acid supplementation for treating RA has been reported^9^. More recent randomized clinical trials indicated that docosahexaenoic acid (DHA) supplementation tended to ameliorate RA disease activity^10^. Of the SPMs, RvD1 and RvD3, both of which are derived from DHA, have been reported to reduce arthritis severity in a K/BxN serum transfer arthritis model, and to be detectable in the synovial fluid of RA patients^11,12^. Other SPMs (RvD5, Maresin 1 (MaR1) and LipoxinA_4_) have also been identified in synovial fluid in RA patients^13^. According to one report, MaR1 decreased arthritis and joint injury scores in a collagen-induced arthritis model^14^.

Several SPMs have been reported to have regulatory effects on the cellular targets that establish the pathology of RA. RvD1, RvD2 and MaR1 suppress Th1 and Th17 polarization and facilitate regulatory T cells (Treg) differentiation of naïve CD4^+^ T cells isolated from human peripheral blood mononuclear cells^15^. The ratio of Treg/Th17 cells is elevated in lymph nodes from MaR1-treated collagen-induced arthritis mice^14^. In addition, RvD1 and RvE1 have been shown to inhibit osteoclastogenesis^16–18^. These reports suggest that RvD1, RvD2, MaR1 and RvE1 are involved in the pathogenesis of RA. However, few reports have examined the role of other SPMs in immune cells and their involvement in inflammatory diseases.

Here we performed LM profiling on inflamed arthritic paws in a mouse model of RA, and found that RvD5 levels were elevated and correlated with arthritis disease activity. We demonstrated that RvD5 suppressed Th17 cells more strongly than RvD1. We also investigated the effects of RvD5 on the different cells involved in the pathology of RA in vitro and in vivo.

## Results

### RvD5 is elevated in the paws of arthritic SKG mice

**Figure 1:**
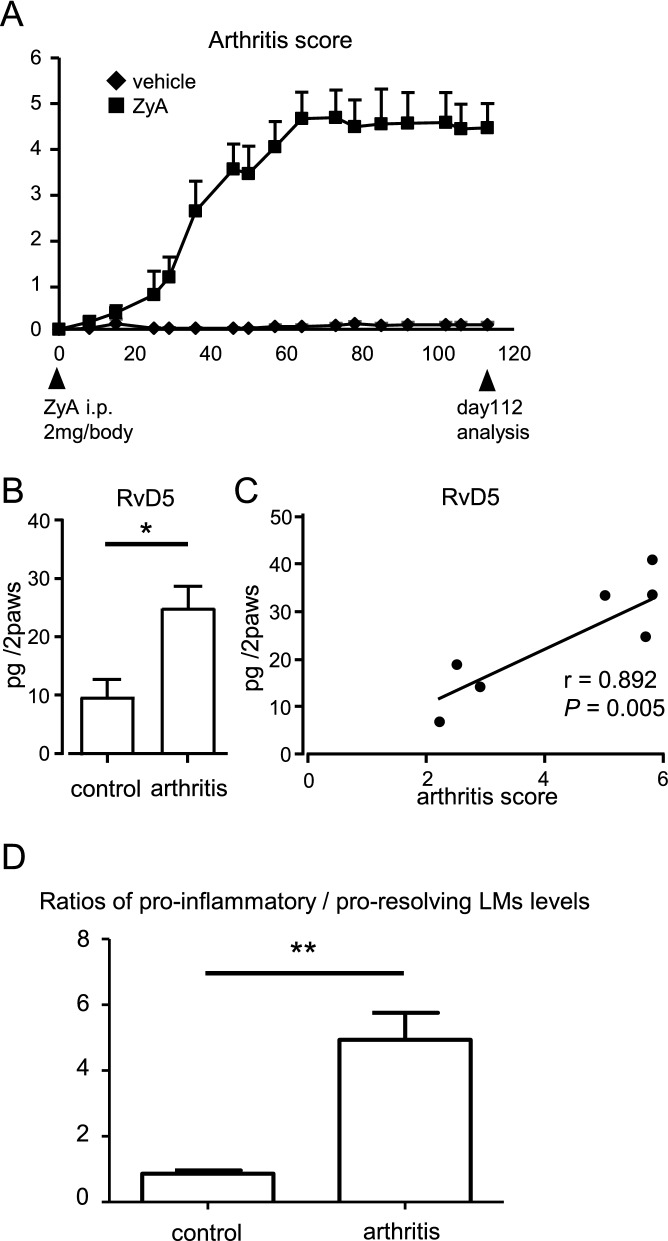
RvD5 is elevated in arthritis and correlates with the degree of arthritis. (**A**) SKG mice were injected with ZyA (2 mg/body i.p.) on day 0. On day 112, paws were removed and analyzed. (**B**) LM quantified using LC/MS/MS-based LM profiling (control group, n = 5, arthritis group, n = 8). Bars represent mean ± SEM. *P < 0.05, by Mann–Whitney U test and FDR-BH correction. (**C**) Correlation between disease activity and RvD5 level (Spearman’s rank correlation coefficient). (**D**) Ratios of pro-inflammatory versus pro-resolving lipid mediator levels. Bars represent mean ± SEM. **P < 0.01, by Mann–Whitney U test.

We injected SKG mice with Zymosan A (ZyA) (n = 8) and observed them for 16 weeks in comparison to mice not receiving ZyA (n = 5). In the arthritic SKG mice, joint inflammation was sustained for these 16 weeks (Fig. 1A). At week 16, we euthanized the mice, collected their paws and used wide-targeted LC/MS/MS-based lipidomics analysis to investigate how LM profiles changed at the inflammatory sites in chronic arthritis (Fig. 1B, Table 1). In arthritic paws, arachidonic acid-derived pro-inflammatory mediators, such as PGE_2_, were significantly elevated relative to non-arthritic paws. Eicosapentaenoic acid (EPA)- or DHA-derived SPMs, such as RvE3, RvD3, RvD5, and MaR2, were also significantly increased in the arthritic paws. RvD1 showed a tendency to be increased (*P* = 0.062). Among these elevated SPMs, levels of RvD5 were most strongly correlated with arthritis disease activity on day 112 (r = 0.892, *P* = 0.005) (Fig. 1C). On day 56, there was also a strong trend for correlation between levels of RvD5 and arthritis score (r = 0.681, P = 0.063) (Fig. S1). These results suggested that RvD5 might be involved in the pathogenesis of arthritis.

**Table 1:** Lipid mediator profiles in arthritic paws of SKG mice.

	Control group	Arthritis group
**AA derived bioactive metabolome**		
Prostaglandin E_2_	6070.3 ± 2198.0	37,005.7 ± 4476.0**
Prostaglandin D_2_	9122.2 ± 2720.1	37,975.4 ± 2616.2**
Prostaglandin J_2_	752.4 ± 118.8	2485.0 ± 291.7**
15-deoxy-Δ^12,14^-Prostaglandin J_2_	19,951.4 ± 3978.8	24,322.9 ± 4456.1
Prostaglandin F_2a_	2013.8 ± 371.0	16,462.7 ± 1812.3**
8iso-Prostaglandin F_2a_	159.7 ± 14.8	617.7 ± 57.1**
Thromboxane B_2_	629.3 ± 195.5	6996.9 ± 854.7**
12-HHT	259.5 ± 33.5	1874.6 ± 165.1**
Leukotriene B_4_	41.0 ± 11.6	165.8 ± 29.7*
Lipoxin A_4_	–	–
Lipoxin B_4_	–	–
5,15-diHETE	8.5 ± 0.6	30.5 ± 4.1**
**AA pathway markers**		
5-HETE	104.4 ± 18.5	464.6 ± 61.3**
12-HETE	1675.0 ± 341.8	6972.2 ± 1017.4**
15-HETE	278.1 ± 39.9	1045.7 ± 134.0**
AA	5927.3 ± 642.6	15,333.5 ± 1019.6**
**EPA derived bioactive metabolome**		
Resolvin E1	ー	34.5 ± 16.9
Resolvin E2	9.0 ± 2.6	8.5 ± 1.7
Resolvin E3	ー	55.4 ± 9.9**
**EPA pathway markers**		
5-HEPE	23.0 ± 4.1	45.8 ± 6.4*
12-HEPE	84.4 ± 13.5	369.8 ± 61.1
15-HEPE	106.0 ± 28.3	208.9 ± 41.5*
18-HEPE	17.0 ± 2.8	53.1 ± 9.3*
EPA	1260.0 ± 106.1	4528.2 ± 630.5**
**DHA derived bioactive metabolome**		
Resolvin D1	13.7 ± 3.3	45.1 ± 9.5
Resolvin D2	ー	ー
Resolvin D3	ー	6.9 ± 1.4*
Resolvin D5	9.6 ± 2.9	24.7 ± 3.7*
Protectin D1	9.3 ± 3.1	10.4 ± 1.3
Maresin1	4.7 ± 2.3	5.9 ± 1.1
Maresin2	52.9 ± 15.7	108.5 ± 13.8*
**DHA pathway markers**		
4-HDHA	45.4 ± 7.5	154.5 ± 19.7**
7-HDHA	10.7 ± 1.5	40.4 ± 5.3**
14-HDHA	350.2 ± 83.8	1210.3 ± 221.0*
17-HDHA	153.5 ± 20.1	510.7 ± 76.7*
DHA	2918.3 ± 269.8	8526.0 ± 612.1**

SKG mice were injected with or without ZyA (2 mg/body i.p.) on day 0. Paws were collected on day 112. LM profiles in paws were determined using LC/MS/MS-based LM profiling (control group, n = 5, arthritis group, n = 8). Values were represented as mean ± SEM.

**P < 0.01, *P < 0.05 by using Mann–Whitney U test and FDR-BH correction.

### Biosynthesis of AA-derived pro-inflammatory mediators might be increased in the arthritic paws

We analyzed the ratio of LMs to precursors to identify whether elevated LMs were actively biosynthesized in arthritic paws. Compared to normal paws, the ratios of arachidonic acid (AA)-derived pro-inflammatory mediators to their precursors were significantly increased (PGF_2a_, and TXB_2_) or tended to be increased (PGE_2_, PGD_2_ and 8iso-PGF_2a_: corrected *P*-value: *P* > 0.05, uncorrected *P*-value: *P* < 0.05 with Mann-whitey U test with FDR-BH correction) in the arthritic paws (Fig. S2). On the contrary, ratio of AA-derived 15-deoxy-Δ^12,14^-Prostaglandin J_2_ to AA, which had been shown to ameliorate arthritis of collagen induced arthritis model^19^, tended to be decreased in the arthritic paws (corrected *P*-value: *P* > 0.05, uncorrected *P*-value: *P* < 0.05). There was no significant difference in EPA-derived and DHA-derived metabolites between arthritic paws and non-arthritic paws, except RvE1, RvE3 and RvD3 which were lower than the detectable range in the non-arthritic paws. These results suggested that the biosynthesis of AA-derived pro-inflammatory mediators might be increased in the arthritic paws.

We also analyzed ratios of pro-inflammatory LMs (PGs + TXB_2_ + LTB_4_) versus pro-resolving (15-deoxy-Δ^12,14^-Prostaglandin J_2_ + Lipoxins + Rvs + PD1 + MaRs) LMs levels to clarify whether the imbalance of pro-inflammatory to pro-resolving LMs contributed to chronic arthritis. The ratios of pro-inflammatory LMs to pro-resolving LMs were significantly higher in arthritic paws than in non-arthritic paws (*P* < 0.01) (Fig. 1D). This result suggested that the insufficiently elevated SPMs and highly elevated pro-inflammatory LMs might be involved in the chronic persistent arthritis of SKG mice.

### RvD1 and RvD5 suppress Th17 cell differentiation and facilitate regulatory T cell differentiation in vitro

**Figure 2:**
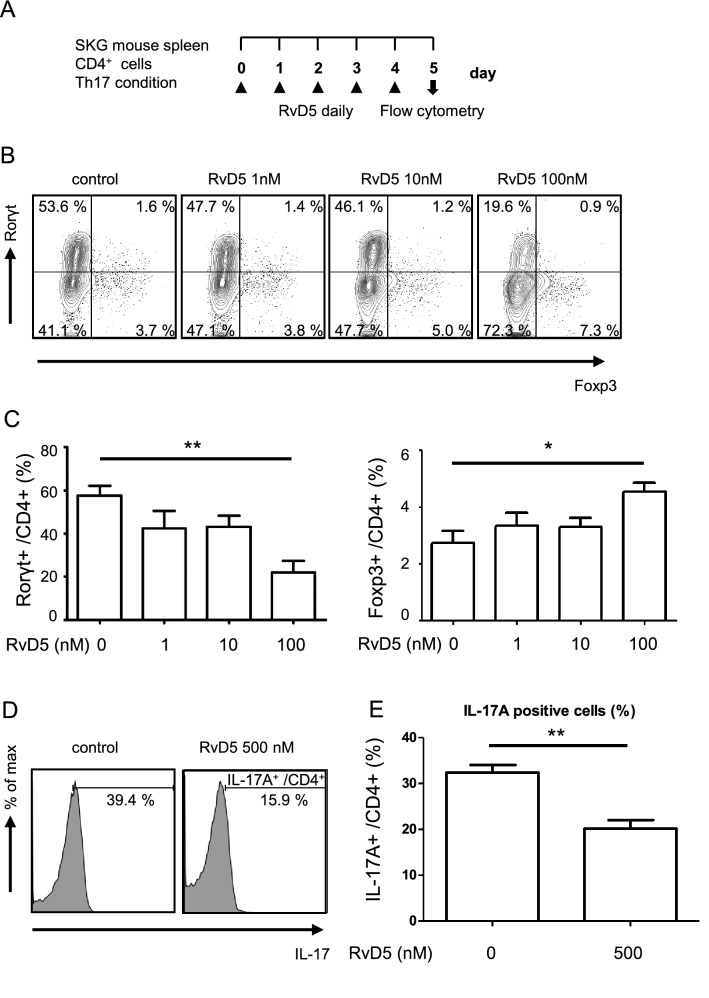
RvD5 suppresses Th17 cell differentiation and facilitates Treg differentiation. (**A**) Schematic representation of the Th17 cell culture conditions and experiment protocol. CD4^+^ T cells were isolated from 3–4 week-old SKG mice and cultured for 5 days in wells pre-coated with anti-CD3 and anti-CD28, in the presence of TGF-β, IL-6, anti-IFNγ antibody, anti-IL-4 antibody, and with or without daily addition of RvD5 (1–100 nM). On day 5, cells were stained with anti-CD4, anti-Rorγt and anti-Foxp3 antibodies. (**B**) Frequencies of Th17 cells (Rorγt^+^/ CD4^+^) and Tregs (Foxp3^+^/ CD4^+^) estimated by flow cytometry. Data are representative of five independent experiments. (**C**) Frequencies of Th17 cells and Tregs in each group, by flow cytometry. (**D**) CD4^+^ T cells were cultured under Th17-inducing conditions for 3 days with or without daily addition of RvD5 (500 nM) and then stimulated with PMA/Ionomycin for 6 h. Cells were stained with anti-CD4, anti-IL-17A antibodies. Frequencies of IL-17A^+^/CD4^+^ cells estimated by flow cytometry. Data are representative of seven independent experiments. (**E**) Frequencies of IL-17A^+^/CD4^+^ cells in each group, by flow cytometry. Bars represent mean ± SEM. **P < 0.01, *P < 0.05, by one-way analysis of variance and Tukey’s multiple comparison test.

Different types of immune cells, such as helper T cells, dendritic cells (DCs), macrophages and OCs, are involved in the pathogenesis of joint inflammation in RA^4^. Therefore, we next examined whether DHA-derived SPMs have any effects on these cells in vitro. First, we cultured CD4^+^ cells with SPMs under Th17-inducing conditions (Fig. 2A, Fig. S3A). Consistent with a previous report, we found that RvD1 inhibited Th17 differentiation and facilitated the differentiation of Tregs^15^ (Fig. S3B, C). Going beyond this, we also found that RvD5 inhibited the differentiation of Rorγt^+^ Th17 cells and increased the population of Foxp3^+^ Tregs (Fig. 2B, C) more potently than RvD1. Thus, while RvD1 caused a 33% reduction of Rorγt^+^ Th17 cells, RvD5 reduced them by 66% at 500 nM. Similarly, RvD1 increased Foxp3^+^ Tregs by 26%, whereas RvD5 resulted in a 39% increase at 500 nM. In contrast, RvD3 and MaR2 did not affect Th17 and Treg differentiation. We also confirmed that RvD5 inhibited the frequency of IL-17A-producing cells (Fig. 2D, E), These results suggest that RvD5 suppresses the differentiation of Th17 cells and increases Tregs more effectively than RvD1.

### RvD5 inhibits CD4^+^ T cell proliferation

**Figure 3:**
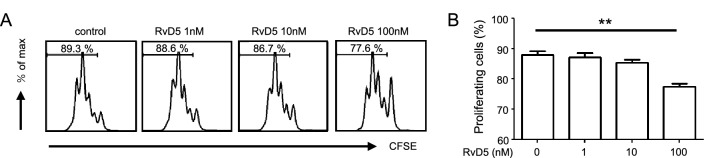
RvD5 suppresses CD4^+^ T cell proliferation. (**A**) CD4^+^ T cell proliferation measured by CFSE fluorescence. Data are representative of five independent experiments. (**B**) Frequencies of proliferating T cells in each group, analyzed by flow cytometry. Bars represent mean ± SEM. **P < 0.01, *P < 0.05, by one-way analysis of variance and Tukey’s multiple comparison test.

A previous report showed that RvD1 did not affect CD4^+^ T cell proliferation^15^. Therefore, we next examined the effect of RvD5 on CD4^+^ T cell proliferation and found that it was in fact suppressed in a dose-dependent manner (Fig. 3A, B). We confirmed that this was not seen for RvD1, and also showed that the same was true for RvD3 or MaR2, which had no effect on CD4^+^ T cell proliferation (Fig. S4). In addition, we studied whether RvD5 affects cell viability under these conditions and found that RvD5 did not affect T cell viability (Fig. S5).

### RvD5 may not affect DC differentiation and activation, or GM-DM phenotypes in vitro

We then examined the effect of RvD5 on myeloid cells. First, we stimulated bone marrow (BM) cells from SKG mice with granulocyte–macrophage colony-stimulating factor (GM-CSF) and IL-4, with or without daily addition of RvD5 (Fig.S6A). We found that RvD5 did not suppress DC differentiation (Fig. S6B). To further explore the effect of RvD5 on DC activation, we incubated DCs with RvD5 before lipopolysaccharide (LPS) stimulation (Fig. S6C) and found that RvD5 had no inhibitory effects on CD86^+^ or MHC class II^+^ populations of CD11b^+^CD11c^+^ cells (Fig. S6D).

To investigate the effect of RvD5 on macrophages, we cultured GM-DMs in an inflammatory environment^20^ and stimulated them with LPS and IFNγ to generate M1 phenotype cells. These were treated or not treated with RvD5 before LPS/IFNγ stimulation (Fig. S6E). There was no effect on macrophage polarization (Fig. S6F). These results suggest that RvD5 may not have any effects on myeloid cells.

### RvD5 decreases bone marrow-derived OC growth and interferes with osteoclastogenesis at the molecular level

**Figure 4:**
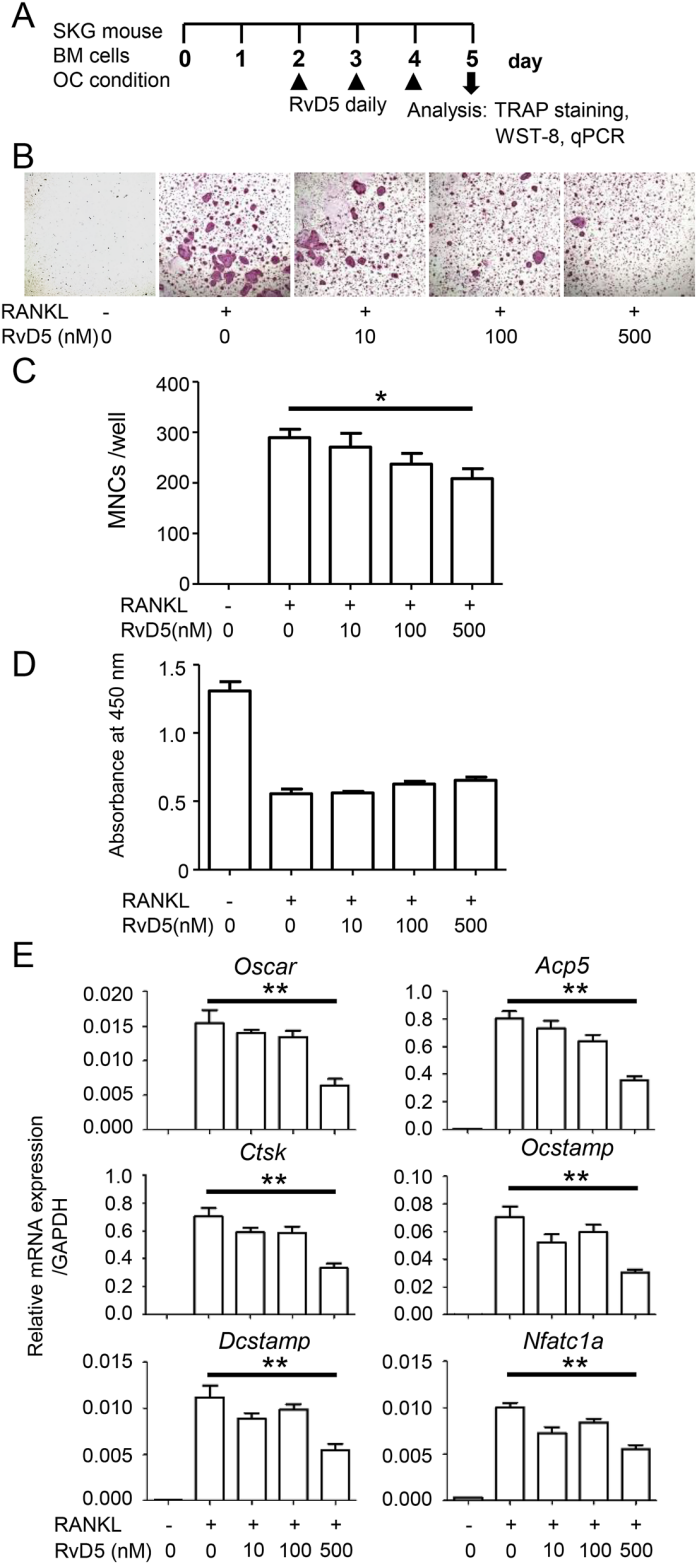
RvD5 suppresses RANKL-induced osteoclastogenesis. (**A**) Schematic representation of RANKL-induced osteoclastogenesis. BM cells were isolated from 8–10-week-old SKG mice and cultured for 2 days with M-CSF. BMMs were then incubated with M-CSF and RANKL, with or without daily RvD5 (10–500 nM). Cells were fixed and stained for TRAP after 5 days of culture. (**B**) Representative images of TRAP staining. (**C**) TRAP-positive MNCs were counted. (**D**) Relative cell viability. (**E**) The mRNA expression levels were quantified by qPCR. Messenger RNA levels were normalized to GAPDH. Bars represent mean ± SEM. **P < 0.01, by one-way analysis of variance and Tukey’s multiple comparison test.

OCs play a pivotal role in the development of bone erosions in RA^21^. We therefore investigated whether RvD5 has any effects on osteoclastogenesis. To this end, we cultured macrophage colony-stimulating factor (M-CSF)-induced bone marrow macrophages (BMMs) stimulated with receptor activator of NF-κB ligand (RANKL) to generate bone marrow-derived OCs, to which RvD5 was added daily (Fig. 4A). RvD5 treatment resulted in decreased formation of RANKL-induced TRAP^+^MNCs in a dose-dependent manner (Fig. 4B, C). We also studied cell viability under these conditions, and found that this was not affected by RvD5 (Fig. 4D). Next, we examined the expression of OC-related genes. Levels of nuclear factor of activated T cells c1a (*NFATc1a*), known as a master transcription factor for OC differentiation, were decreased by RvD5 treatment in a dose-dependent manner (Fig. 4E). Many other genes associated with osteoclastogenesis, such as osteoclast-associated receptor (*Oscar*), acid phosphatase 5 (*Acp5*), cathepsin K (*Ctsk*), osteoclast stimulatory transmembrane protein (*Ocstamp*), dendritic cell-specific transmembrane protein (*Dcstamp*), were also suppressed by RvD5 treatment. These results suggest that RvD5 may be involved in the pathogenesis of RA by inhibiting the differentiation of OCs.

### RvD5 treatment does not prevent arthritis in the SKG model in vivo

Because RvD5 suppressed Th17 cell differentiation and facilitated Treg differentiation, inhibited CD4^+^ T cell proliferation and interfered with osteoclastogenesis, we hypothesized that RvD5 treatment might prevent arthritis progression in SKG mice. We administered RvD5 (1000 ng daily) or a physiological saline solution intraperitoneally to ZyA-induced SKG mice. RvD5 treatment did not prevent arthritis in the SKG model in vivo (Fig. S7).

## Discussion

In the present study, we demonstrated that both pro-inflammatory mediators and SPMs are elevated in the arthritic paws in SKG mice, a model of chronic arthritis. Two recent studies have reported LC/MS/MS-based LM metabolomics analysis on arthritic paws in other model of arthritis^11,12^. Both studies used a K/BxN serum transfer model, which exhibits self-limited arthritis peaking at around 10 days. Levels of many pro-inflammatory mediators tended to increase during the inflammatory phase (days 4–8), and decrease again in the resolution phase (day 16). In contrast, levels of several SPMs including D-series resolvins were decreased during the inflammatory phase and peaked in the resolution phase. This study is the first to perform LC/MS/MS-based LM metabolomics analysis in a chronic persistent arthritis model and we found that, in the chronic course, levels of many pro-inflammatory mediators remained very high and the ratios of pro-inflammatory to pro-resolving LMs levels were significantly higher in arthritic paws than in non-arthritic paws. Our results are consistent with a recent study showing that serum MaR1 levels in active RA patients are significantly lower than in inactive RA patients^14^. These findings suggest that persistent high levels of pro-inflammatory mediators and insufficiently elevated SPMs contribute to the pathology of chronic joint inflammation.

Jonasdottir et al.^22^ investigated lipid mediators in synovial fluid from osteoarthritis and RA patients by LC/MS/MS analysis. The authors showed that AA, EPA, DHA and these metabolites were detectable in RA, and concentration ratios of several AA-derived metabolites to their precursors were higher in RA than in osteoarthritis. The results in human RA study are consistent with our findings showing that concentration ratios of AA-derived pro-inflammatory mediators/AA were increased in mouse model of RA. These results suggest that pro-inflammatory mediators may be actively biosynthesized in the arthritic joints.

RvD5 was initially identified in media from ionophore-stimulated trout brain cells^23^. Previous studies have demonstrated that RvD5 is produced by M2 macrophages and has an enhancing effect on their phagocytosis^24,25^. A previous report showed that RvD5 is present in synovial fluid from patients with RA^13^, but its role in the pathogenesis of this disease is unknown. To the best of our knowledge for the first time, we here report that RvD5 suppressed Th17 cell differentiation and facilitated Treg differentiation. Higher percentages of circulating Th17 cells and higher concentrations of IL-17A have been reported in sera from RA patients^26,27^. A Th17/Treg imbalance has been suggested to be involved in the pathogenesis of RA^26^. Our findings imply that increasing RvD5 levels might be able to correct Th17/Treg cell imbalances and suppress joint inflammation in RA.

RvD1 has been reported to inhibit bone resorption in a murine model of collagen antibody-induced arthritis^18^. A previous study showed that DHA exerted an inhibitory effect on OC formation which was counteracted by 5-lipoxygenase inhibition^28^. In the present study we demonstrated that RvD5 inhibits OC differentiation. Because 5- lipoxygenase acts as an essential enzyme to convert DHA to D-series resolvins, RvD5, in addition to other D-series resolvins, may suppress bone destruction in RA by suppressing OC differentiation.

In vivo RvD5 treatment did not prevent arthritis in this model. There are several possible reasons why this was the case. PGE_2_ has been reported to promote Th17 cell differentiation and OC maturation^29^. LTB_4_ induces IL-1 and TNF production by RA fibroblast-like synoviocytes and promotes neutrophil, macrophage and CD4^+^ T cell recruitment into the joint^30^. Because we measured extremely high levels of PGE_2_ and LTB_4_ in the inflammatory joints in SKG mice, high levels of pro-inflammatory SPMs might be the reason for the lack of a significant effect of RvD5 monotherapy in vivo. In addition, weak effects of RvD5 on DCs and macrophages might contribute to the result. Previous study demonstrated that RvD5 down-regulated genes of inflammatory markers in macrophages^15^. As for the effect of RvD5 on myeloid cells in this study, we might have underestimated its effect because serum (1% FBS) in the medium might affect the effects of SPMs on target cells. Also, standard deviation of in vivo experiment was much higher than we expected.

In conclusion, our results suggest that elevation of RvD5 is not simply a result of inflammation, but is involved in the pathogenesis of RA. Targeting the resolution of inflammation seems promising as a novel treatment of RA.

## Conclusions

SPMs including RvD5 were found to be elevated in localized inflammation in animals with chronic arthritis. RvD5 suppressed Th17 cell differentiation and CD4^+^ T cell proliferation, facilitated Treg differentiation, and suppressed osteoclastogenesis. Further investigations are required to determine the effect of SPM on chronic arthritis and to determine cell targets in RA pathogenesis.

## Methods

### Ethical provisions

This study was approved by the President of Kobe University after the review by Institutional Animal Care and Use Committee (Permission number: P 170703) and carried out according to the Kobe University Animal Experimentation Regulations. This study was carried out in compliance with the ARRIVE guidelines.

### Animals

SKG mice were purchased from CLEA Japan, Inc. They were housed in the Kobe University animal facility. All procedures were carried out in accordance with the recommendations of the Institutional Animal Care Committee of Kobe University.

### Reagents and antibodies

RvD1, RvD3, RvD5 and MaR2 were purchased from Cayman Chemical; ZyA from Alfa Aesar or Sigma-Aldrich; RPMI 1640 medium from Wako Pure Chemical Industries; MEMα from Gibco; fetal bovine serum (FBS) from MP Biomedicals; penicillin/streptomycin mixed solution (P/St) from nacalai tesque; 2-mercaptoethanol (2-ME) and LPS from Sigma-Aldrich; recombinant murine IL-6, murine GM-CSF, murine M-CSF and murine sRANKL from PeproTech; recombinant mouse IL-4 protein, murine transforming growth factor-β (TGF-β), murine IFN-γ, mouse IL-4 antibody and mouse IFN-γ antibody from R&D systems; Purified NA/LE Hamster anti-mouse CD3e and CD28 from BD Pharmingen; 5,6-carboxyfluorescein diacetate N-succinimidyl ester (CFSE-DA) Cell Proliferation Kit from Invitrogen; cell staining buffer and True-NuclearTM Transcripition Factor Buffer Set from BioLegend. Purified rat anti-mouse CD16/CD32 (mouse Fc block) from BD Pharmingen.

Fluorescein isothiocyanate (FITC)-conjugated anti-CD86, FITC-conjugated anti-CD4, FITC-conjugated anti-rat IgG2a kappa isotype, FITC-conjugated anti-rat IgG2b kappa isotype, Allophycocyanin (APC)-conjugated anti-CD11c, APC-conjugated anti-Rorγt, APC-conjugated anti-F4/80, APC-conjugated anti-IL-17A, APC-conjugated anti-mouse IgG1 kappa isotype, phycoerythrin (PE)-conjugated anti-MHC class II, PE-conjugated Foxp3, PE-conjugated anti-CD206, and PE-conjugated anti-rat IgG2a kappa isotype were obtained from Invitrogen. Peridinin-Chlorophyll-Protein (PerCP)-conjugated anti-CD11b and PerCP-conjugated anti-rat IgG2b kappa isotype were purchased from BioLegend.

### SKG arthritis model

Arthritis was induced in 8–10 weeks-old SKG mice by the injection of 2 mg/body ZyA intraperitoneally, as previously described^31^. The development and severity of arthritis was monitored using a previously described system for scoring clinical arthritis^32^. At 56 days (8 weeks) or 112 days (16 weeks) after ZyA injection, control and ZyA injected mice were sacrificed and paws collected (N = 5–8). For LM profiling, we used LC/MS. For in vivo RvD5 treatments, daily RvD5 or a physiological saline solution was administered intraperitoneally.

### LC/MS/MS-based LM lipidomics analysis

Deuterated internal standards d_4_-LTB_4_, d_8_-5-HETE, d_4_-PGE_2_, and d_5_-RvD2 representing each chromatographic region of identified LMs were added to the samples (500 pg each) to facilitate quantification. Samples were extracted by SPE on C18 columns as previously described^33^. They were then subjected to LC/MS/MS using a Q-Trap 6500 (Sciex) equipped with a Shimadzu LC-30AD HPLC system. A ZORBAX Eclipse Plus C18 column (100 mm × 4.6 mm, 3.5 μm, Agilent Technologies) was used with a methanol/water/acetic acid gradient of 55:45:0.01 to 98:2:0.01 (v/v/v) at a 0.4 ml/min flow rate. For monitoring and quantifying the levels of targeted LMs, a multiple reaction monitoring (MRM) method was developed with signature ion pairs Q1 (parent ion)/Q3 (characteristic fragment ion) for each molecule. Identification was based on published criteria using the LC retention time, specific fragmentation patterns, and at least six diagnostic fragmentation ions. Quantification was carried out on the basis of peak area of the MRM chromatograph, and linear calibration curves were obtained with authentic standards for each compound.

### CD4^+^ T cell isolation

SKG mice (3–4 weeks old) were sacrificed and splenocytes isolated after erythrocyte lysis using ACK Lysing Buffer. To isolate CD4^+^ T cells from single cell suspensions prepared from the splenocytes, we used a biotinylated mAb against CD4, streptavidin-coated magnetic beads and a manual MACS system (all from Miltenyi Biotec) according to the manufacturer’s protocol.

### Th17 cell differentiation

Isolated CD4^+^ T cells (2.0 × 10^5^/well) were cultured for 3 days in RPMI supplemented with 1%FBS, 1% P/St and 50 µM 2-ME under the following conditions: on day -1, 96-well plates were pre-coated with 10 ng/ml anti-CD3 mAbs and 5 ng/ml anti-CD28 mAbs, and cells were cultured with 20 ng/ml IL-6, 0.5 ng/ml TGF-β, 2.5 μg/ml anti-IFN-γ and 2.5 μg/ml anti-IL-4 Abs (R&D systems). RvD1, D3, D5 or MaR2 (1, 10, 100, or 500 nM) were added daily from day 0. On day 3 or 5, cells were analyzed by flow cytometry.

### T cell proliferation

CD4^+^ T cells were incubated with 10 mM CFSE according to the manufacturer’s protocol and were cultured in RPMI supplemented with 1% FBS, 1% P/St and 50 µM 2-ME for 3 days. RvD5 (1, 10, or 100 nM) was added daily from day 0. On day 3, cells were analyzed by flow cytometry.

### Cell viability assay for T cell

Cell viability were analyzed using the annexin-V-FLUOS staining Kit (Roche, USA) according to the manufacturer’s protocol. Healthy cells were detected by staining the cells with annexin V and propidium iodide solution and analyzed by flow cytometry.

### Dendritic cell differentiation and activation

SKG mice (8–12 weeks old) were euthanized and BM cells were isolated from the femurs. For the DC differentiation experiments, BM cells (1.0 × 10^6^/well) were cultured for 5 days in RPMI 1640 supplemented with 1% FBS, 1% P/St and 50 µM 2- ME containing GM-CSF (20 ng/ml) and IL-4 (10 ng/ml) at 37 °C in 5% CO_2_. RvD1 (100 nM) and RvD5 (1, 10, or 100 nM) were added daily for 5 days. For DC activation, cells were cultured with LPS (1 μg/ml) and treated with RvD5 (1, 10, 100, or 1000 nM) on day 5. On day 6, cells were evaluated by flow cytometry.

### GM-CSF-induced bone marrow macrophages (GM-DMs) and M1 polarization

BM cells (1.0 × 10^6^/well) were cultured for 3 days in RPMI 1640 containing GM-CSF (10 ng/ml). On day 4, non-adherent cells were collected and reseeded into 12-well plates at 2.0 × 10^6^/well with GM-CSF. On day 7, at which time we considered these cells to be GM-DMs, the medium was changed, and cells were stimulated with LPS (100 ng/ml) and IFN-γ (50 ng/ml) for M1 polarization. On day 8, adherent cells were collected and cultured in 48-well plates in RPMI 1640 supplemented with 1% FBS and RvD5 (1, 10, 100, or 1000 nM). After 6 h of RvD5 treatment, cells were evaluated by flow cytometry.

### Flow cytometry

For surface staining, cells were washed with cell-staining buffer and incubated with anti-CD4, anti-CD11b, anti-CD11c, anti-F4/80, anti-CD86, anti-MHC class II and anti-CD206 antibodies for 30 min at 4 °C. For intracellular staining CD4^+^ T cells were treated with the nuclear factor fixation and permeabilization buffer and stained with anti-Rorγt and anti-Foxp3 antibodies, according to the manufacturer’s protocol. For cytokine staining, cultured CD4^+^ T cells were stimulated in culture medium containing 10 ng/ml PMA and 1 ng/ml Ionomycin for 6 h, with 3 µg/ml brefeldin for last 2 h. All samples were measured on a FACS Verse (BD Bioscience) and analyzed with FlowJo software (Tree Star).

### Osteoclast differentiation

Mouse BM cells were cultured on Petri dishes. After 3 h, non-adherent cells were collected and seeded into 48-well plates at 5.0 × 10^4^/well or into 96-well plates at 2.0 × 10^4^/well in MEMα supplemented with 10% FBS, 1% P/St, and M-CSF (25 ng/ml). After preculture for 2 days, BMs were stimulated with M-CSF and RANKL (50 ng/ml). After a further 3 days, RvD5 (10, 100, or 500 nM) was added daily and cells were collected on day 5. Tartrate-resistant acid phosphatase-positive multinucleated cells (TRAP^+^ MNCs; ≥ 3 nuclei) were visualized using a TRAP staining kit (Cosmo Bio Co., Ltd.) according to the manufacturer’s protocol, and OCs were counted under a microscope (Keyence). For the detection of relative cell viability in osteoclastogenesis, we used Cell Counting Kit (Dojindo) according to the manufacturer’s protocol. After 3 h, results were obtained by a microplate reader at 450 nm.

### Quantitative RT-PCR

Total RNA was extracted using RNeasy Mini kits (Qiagen) and complementary DNA was synthesized by a QuantiTect Reverse Transcription Kit (Qiagen). Quantitative RT-PCR was performed using a QuantiTect SYBR Green PCR kit (Qiagen) with PikoReal system (Thermo Fisher Scientific). GAPDH mRNA was used for internal normalization.

### Statistical analysis

Results were expressed as means and SEMs. Statistical analysis of LM levels was performed by Mann–Whitney U test with false-discovery rate (FDR)-BH correction. Correlations between disease activity and RvD5 levels were estimated by the Spearman’s rank correction coefficient procedure. For comparisons of three or more groups, one-way ANOVA and Tukey’s multiple comparison test were performed using GraphPad Prism 5 (GraphPad Software). *P* < 0.05 was considered statistically significant.

## Supplementary Information

Supplementary Information.

## References

[1] Serhan CN, Savill J (2005). Resolution of inflammation: the beginning programs the end. Nat. Immunol..

[2] Buckley CD, Gilroy DW, Serhan CN, Stockinger B, Tak PP (2013). The resolution of inflammation. Nat. Rev. Immunol..

[3] Serhan CN (2014). Pro-resolving lipid mediators are leads for resolution physiology. Nature.

[4] Buckley CD, Gilroy DW, Serhan CN (2014). Proresolving lipid mediators and mechanisms in the resolution of acute inflammation. Immunity.

[5] Müller-Ladner U, Pap T, Gay RE, Neidhart M, Gay S (2005). Mechanisms of disease: the molecular and cellular basis of joint destruction in rheumatoid arthritis. Nat Clin Pract Rheumatol..

[6] Firestein GS, McInnes IB (2017). Immunopathogenesis of rheumatoid arthritis. Immunity.

[7] Shinohara M, Serhan CN (2016). Novel endogenous proresolving molecules: essential fatty acid-derived and gaseous mediators in the resolution of inflammation. J Atheroscler Thromb..

[8] Mclnnes IB, Schett G (2011). The pathogenesis of rheumatoid arthritis. N Engl J Med..

[9] Goldberg RJ, Katz J (2007). A meta-analysis of the analgesic effects of omega-3 polyunsaturated fatty acid supplementation for inflammatory joint pain. Pain.

[10] Dawczynski C (2018). Docosahexaenoic acid in the treatment of rheumatoid arthritis: A double-blind, placebo-controlled, randomized cross-over study with microalgae vs. sunflower oil. Clin Nutr..

[11] Norling VL (2016). Proresolving and cartilage-protective actions of resolvin D1 in inflammatory arthritis. JCI Insight..

[12] Arnardottir HH (2016). Resolvin D3 is dysregulated in arthritis and reduces arthritic inflammation. J Immunol..

[13] Giera M (2012). Lipid and lipid mediator profiling of human synovial fluid in rheumatoid arthritis patients by means of LC-MS/MS. Biochim Biophys Acta..

[14] Jin S (2018). Maresin 1 improves the Treg/Th17 imbalance in rheumatoid arthritis through miR-21. Ann Rheum Dis..

[15] Chiurchiù V (2016). Proresolving lipid mediators resolvin D1, resolvin D2, and maresin 1 are critical in modulating T cell responses. Sci Transl Med..

[16] Herrera BS (2008). An endogenous regulator of inflammation, resolvin E1, modulates osteoclast differentiation and bone resorption. Br J Pharmacol..

[17] Zhu M, Van Dyke TE, Gyurko R (2013). Resolvin E1 regulates osteoclast fusion via DC-STAMP and NFATc1. FASEB J..

[18] Benabdoun HA (2019). In vitro and in vivo assessment of the proresolutive and antiresorptive actions of resolvin D1: relevance to arthritis. Arthritis Res Ther..

[19] Carregaro V (2016). Therapeutic treatment of arthritic mice with 15-Deoxy Δ^12,14^-prostaglandin J_2_ (15d-PGJ_2_) ameliorates disease through the suppression of Th17 cells and the induction of CD4^+^CD25^-^FOXP3^+^ cells. Mediators Inflamm..

[20] Becher B, Tugues S, Greter M (2016). GM-CSF: from growth factor to central mediator of tissue inflammation. Immunity.

[21] Schett G, Hayer S, Zwerina J, Redlich K, Smolen JS (2005). Mechanisms of Disease: the link between RANKL and arthritic bone disease. Nat Clin Pract Rheumatol..

[22] Jónasdóttir HS (2017). Targeted lipidomics reveals activation of resolution pathways in knee osteoarthritis in humans. Osteoarthritis Cartilage.

[23] Hong S (2005). Rainbow trout (Oncorhynchus mykiss) brain cells biosynthesize novel docosahexaenoic acid-derived resolvins and protectins-Mediator lipidomic analysis. Prostaglandins Other Lipid Mediat..

[24] Chiang N (2012). Infection regulates pro-resolving mediators that lower antibiotic requirements. Nature.

[25] Werz O (2018). Human macrophages differentially produce specific resolvin or leukotriene signals that depend on bacterial pathogenicity. Nat Commun..

[26] Samson M (2012). Brief report: inhibition of interleukin-6 function corrects Th17/Treg cell imbalance in patients with rheumatoid arthritis. Arthritis Rheum..

[27] Shen H, Goodall JC, Hill Gaston JS (2009). Frequency and phenotype of peripheral blood Th17 cells in ankylosing spondylitis and rheumatoid arthritis. Arthritis Rheum..

[28] Yuan J (2010). The effects of polyunsaturated fatty acids and their metabolites on osteoclastogenesis in vitro. Prostaglandins Other Lipid Mediat..

[29] Samuels JS (2018). Shashidharamurthy, prostaglandin E2 and IL-23 interconnects STAT3 and RoRγ pathways to initiate Th17 CD4^+^ T-cell development during rheumatoid arthritis. Inflamm Res..

[30] Miyabe Y, Miyabe C, Luster AD (2017). LTB_4_ and BLT1 in inflammatory arthritis. Semin Immunol..

[31] Yoshitomi H (2005). A role for fungal {beta}-glucans and their receptor Dectin-1 in the induction of autoimmune arthritis in genetically susceptible mice. J Exp Med..

[32] Nishimura K, Saegusa J, Matsuki F, Akashi K, Kageyama G, Morinobu A (2015). Tofacitinib facilitates the expansion of myeloid-derived suppressor cells and ameliorates arthritis in SKG mice. Arthritis Rheumatol..

[33] Colas RA, Shinohara M, Dalli J, Chiang N, Serhan CN (2014). Identification and signature profiles for pro-resolving and inflammatory lipid mediators in human tissue. Am J Physiol Cell Physiol..

