# Supplementary material for: Effect of resolvin D5 on T cell differentiation and osteoclastogenesis analyzed by lipid mediator profiling in the experimental arthritis
Source: Sci Rep. 2021 Aug 27;11:17312. doi: 10.1038/s41598-021-96530-1 (PMC8397777; doi:10.1038/s41598-021-96530-1)
Supplement: Supplementary file 1 — Supplementary Information. [file 41598_2021_96530_MOESM1_ESM.pdf]

Effect of resolvin D5 on T cell differentiation and osteoclastogenesis analyzed by lipid mediator profiling in the experimental arthritis

Hirotaka Yamada,[1] Jun Saegusa,[1 2] Sho Sendo,[1] Yo Ueda,[1] Takaichi Okano,[1 2] Masakazu Shinohara,[3 4] Akio Morinobu[1]

[1] Department of Rheumatology and Clinical Immunology, Kobe University Graduate School of Medicine, Kobe, Japan.

[2] Department of Clinical Laboratory, Kobe University Hospital, Kobe, Japan.

[3] Division of Epidemiology, Kobe University Graduate School of Medicine, Kobe, Japan.

[4] The Integrated Center for Mass Spectrometry, Kobe University Graduate School of Medicine, Kobe, Japan.

Supplemental figure 1

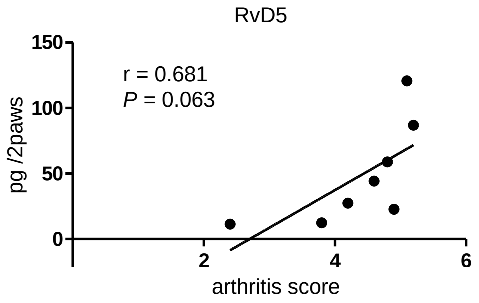

Supplemental figure 1. Levels of RvD5 tend to be correlated with arthritis severity on day 56. SKG mice were injected with ZyA (2 mg/body i.p.) on day 0. Eight weeks after ZyA injection (on day 56), paws were removed and LM quantified using LC/MS/MS-based LM profiling (control group, n = 5, arthritis group, n = 8). Correlation between disease activity and RvD5 level was analyzed by Spearman's rank correlation coefficient.

Supplemental figure 2

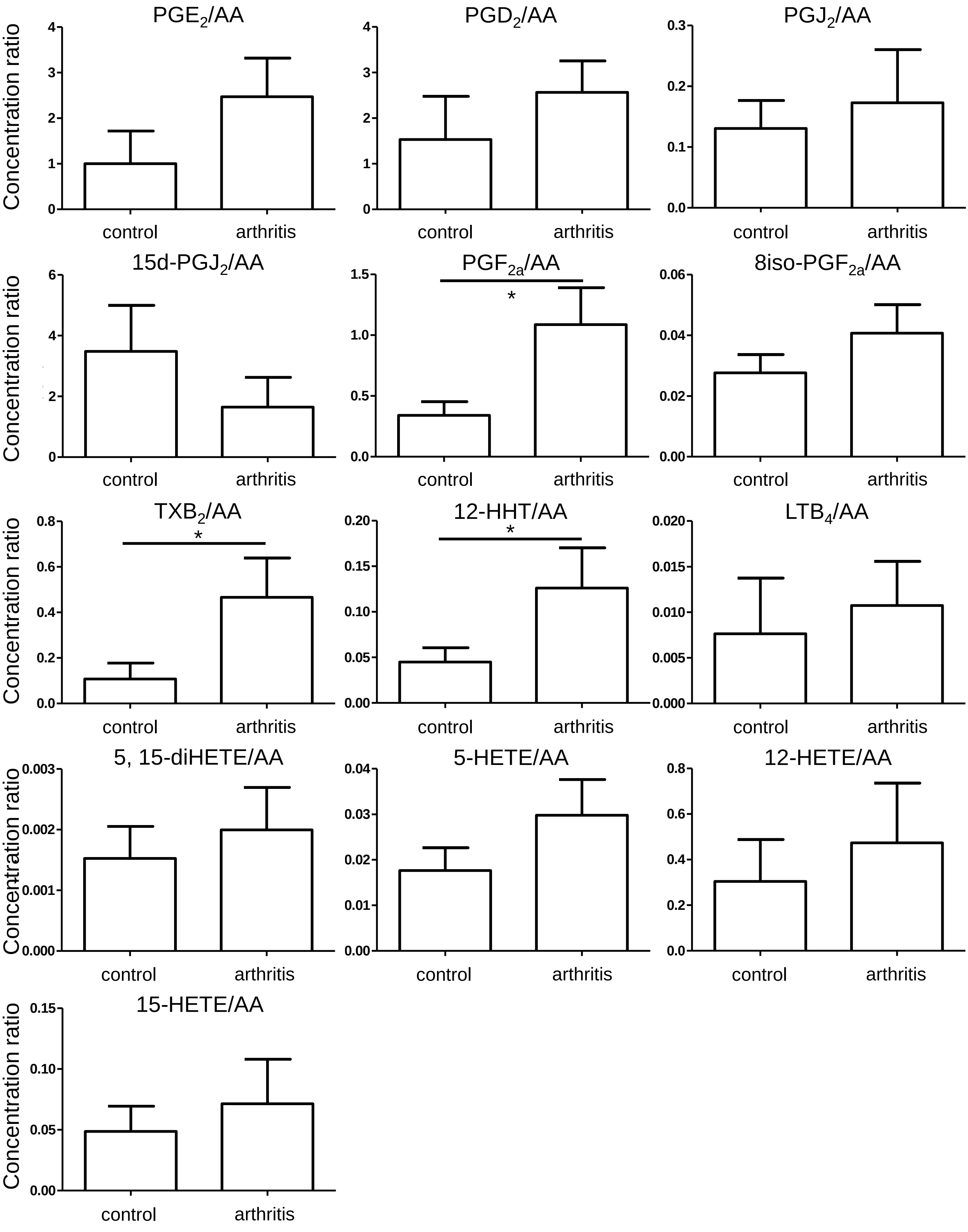

Supplemental figure 2. Biosynthesis of AA-derived pro-inflammatory mediators might be increased in the arthritic paws.

SKG mice were injected with ZyA (2 mg/body i.p.) on day 0. On day 112, paws were removed and LM quantified using LC/MS/MS-based LM profiling (control group, n = 5, arthritis group, n = 8). Concentrations ratios of LMs to their precursors. Bars represent mean ± SEM. **P* < 0.05, by Mann-Whitney U tests and FDR-BH correction.

Supplemental figure 2 (continue)

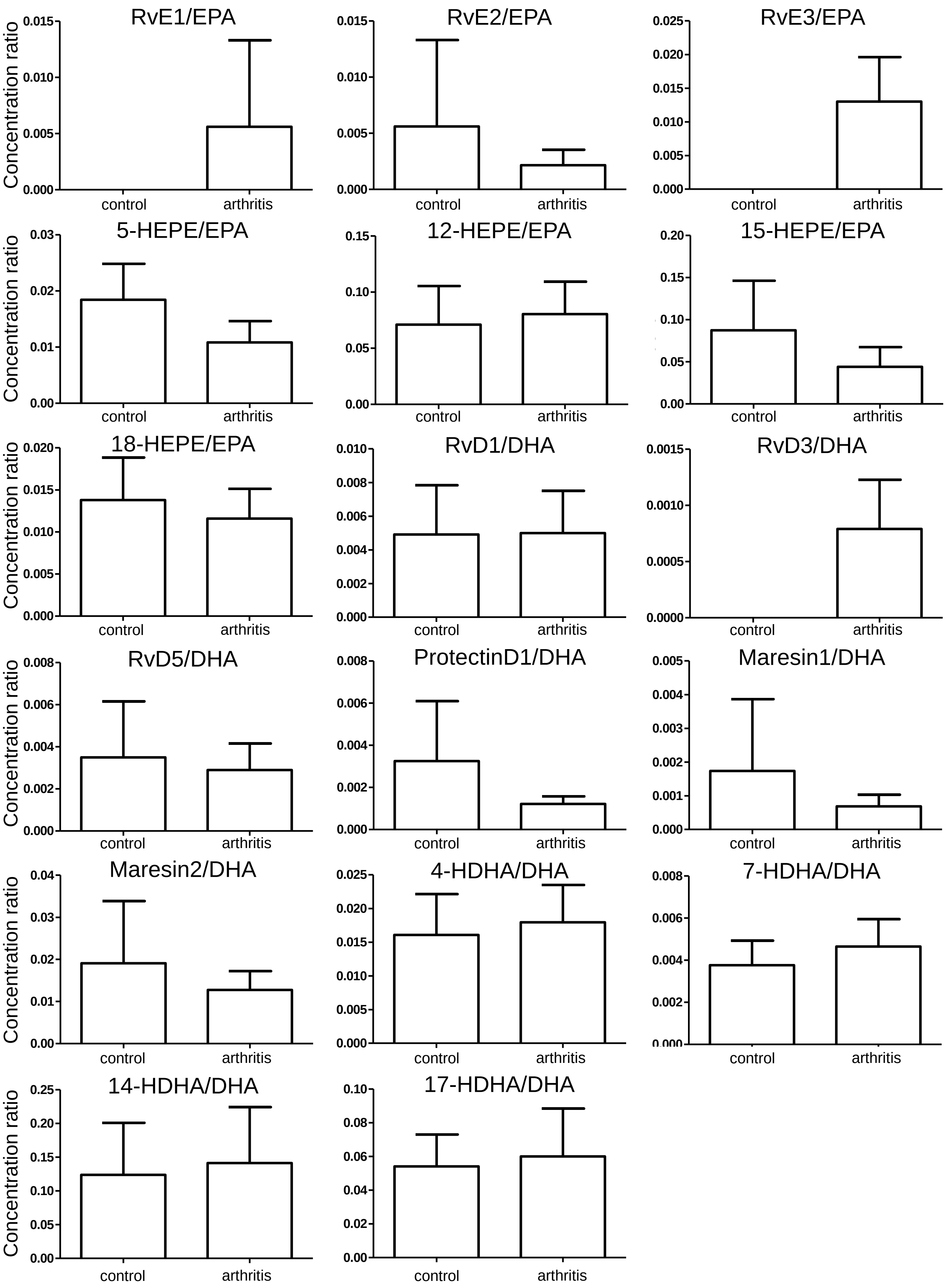

Supplemental figure 3

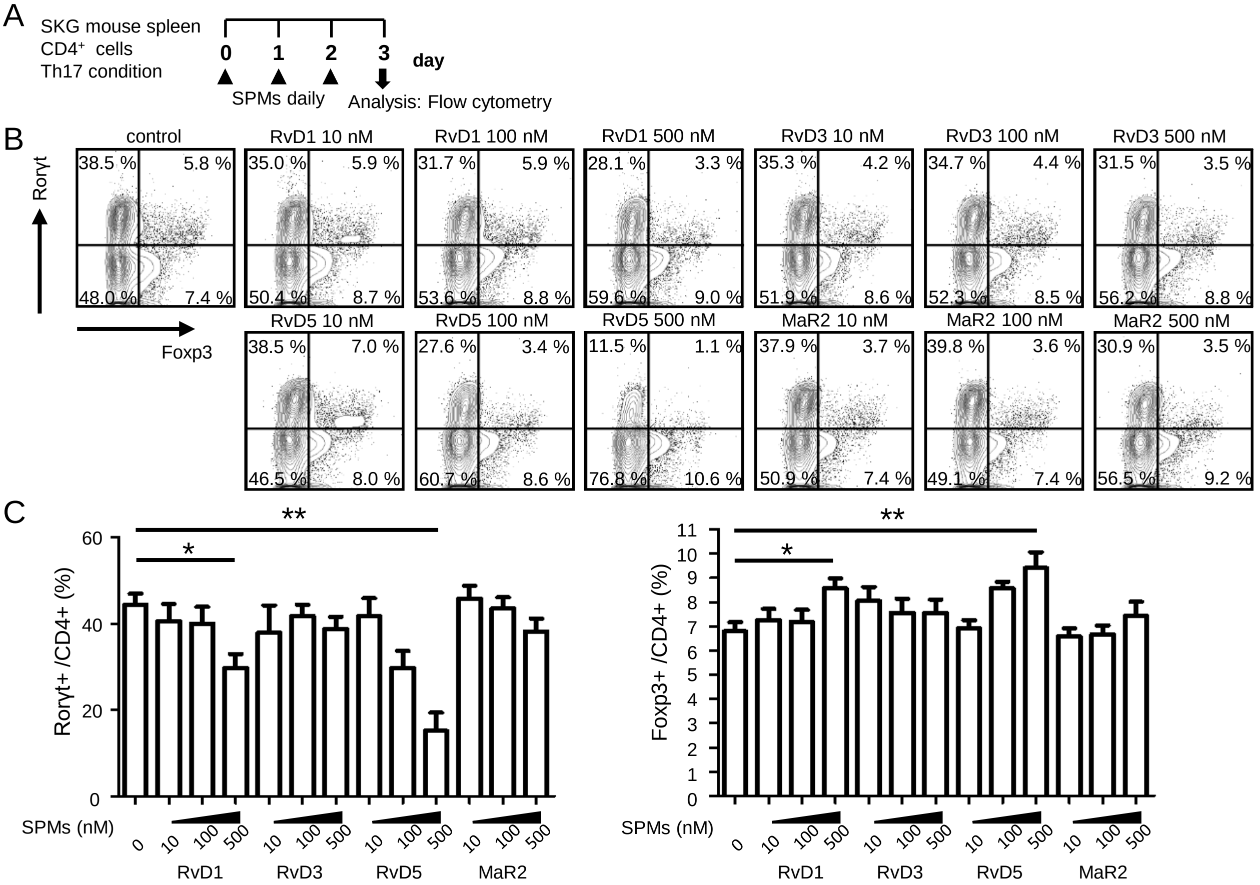

Supplemental figure 3 RvD1 and RvD5 suppresses Th17 cell differentiation and facilitates Treg differentiation.

(A) Schematic representation of the Th17 cell culture conditions and experiment protocol. CD4+ T cells were isolated from 3-4 week-old SKG mice and cultured for 3 days in wells pre-coated with anti-CD3 and anti-CD28, in the presence of TGF-β, IL-6, anti-IFNγ antibody, anti-IL-4 antibody, and with or without daily addition of SPMs (10-500 nM). On day 3, cells were stained with anti-CD4, anti-Rorγt and anti-Foxp3 antibodies. (B) Frequencies of Th17 cells (Rorγt$^+$/ CD4$^+$) and Tregs (Foxp3$^+$/ CD4$^+$) estimated by flow cytometry. Data are representative of five independent experiments. (C) Frequencies of Th17 cells and Tregs in each group, by flow cytometry. Bars represent mean ± SEM. **P < 0.01, *P < 0.05, by one-way analysis of variance and Tukey’s multiple comparison test.

Supplemental figure 4

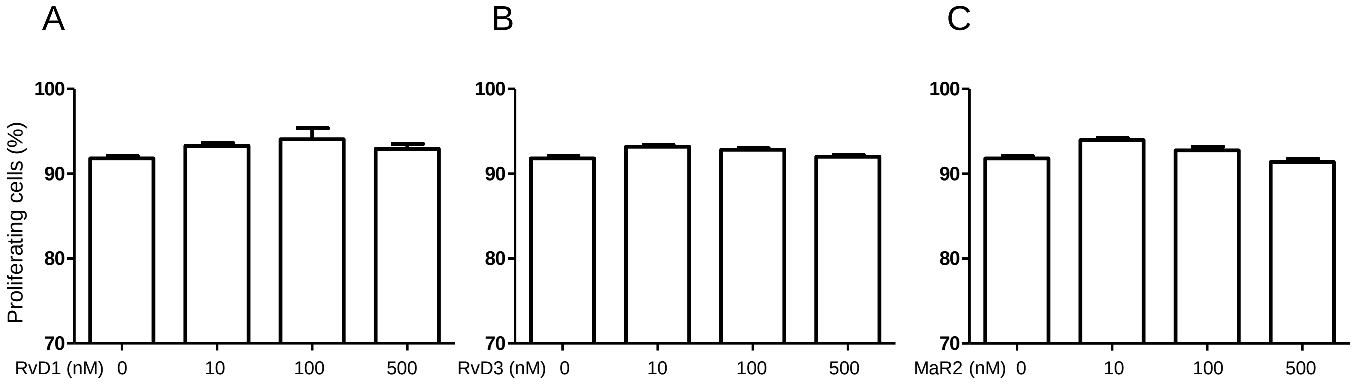

Supplemental figure 4. RvD1, RvD3 and MaR2 do not suppress CD4⁺ T cell proliferation. Frequencies of proliferating T cells in each group (n = 4). CD4⁺ T cell were isolated from 3-4 week old SKG mice, incubated with CFSE and cultured for 3days in wells pre-coated with anti-CD3 and anti-CD28 antibody, and with or without daily addition of SPMs (**(A)** RvD1, **(B)** RvD3, and **(C)** MaR2) (10-500nM). On day 3, cell were stained with anti-CD4 and analyzed by flow cytometry.

Supplemental figure 5

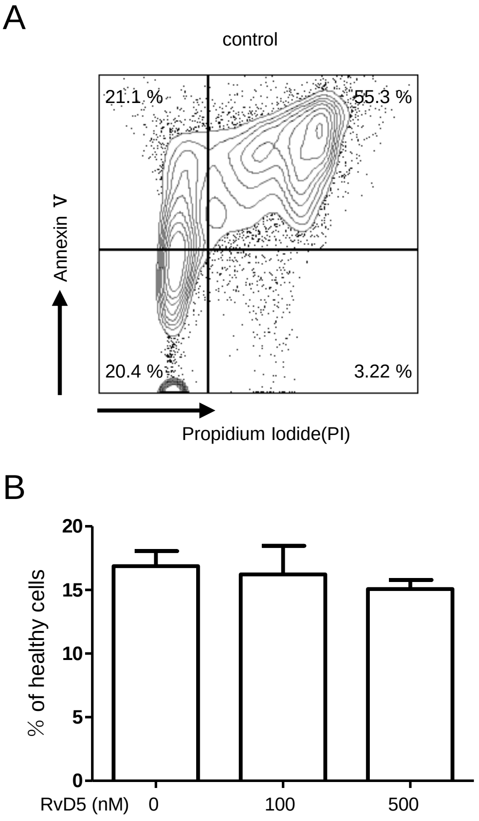

Supplemental figure 5. RvD5 does not effect cell viability.
(A) CD4+ T cell were isolated from 3-4 week old SKG mice, and cultured for 3days in wells pre-coated with anti-CD3 and anti-CD28 antibody, and with or without daily addition of SPMs (100, 500nM). Cell viability were analyzed using the annexin-V-FLUOS staining Kit (Roche, USA) according to the manufacturer’s protocol. **(B)** Healthy cells and necrotic cells were detected by staining the cells with annexin V and propidium iodide solution followed by flow cytometry. Data are representative of four independent experiments.

Supplemental figure 6

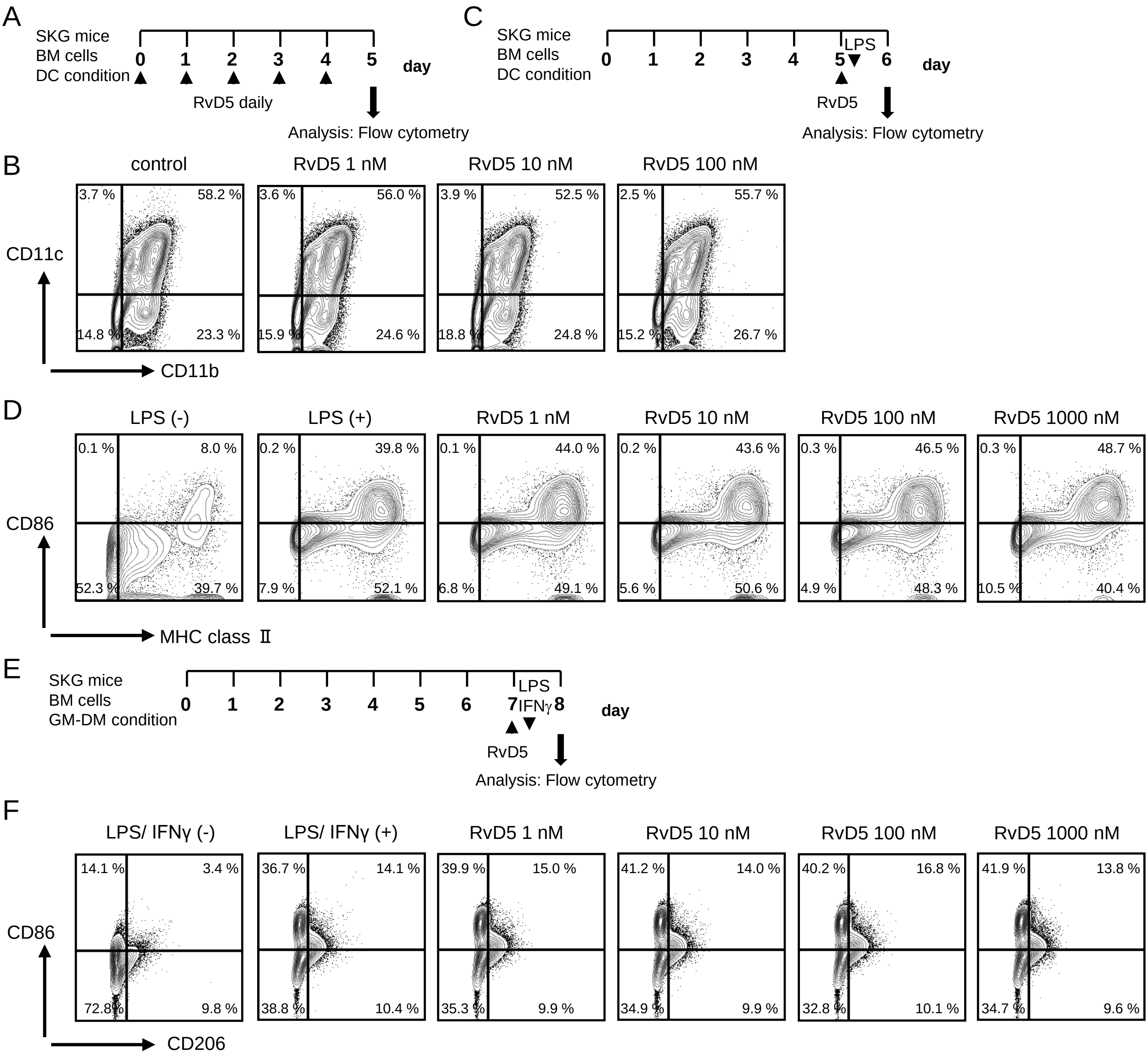

Supplemental figure 6. RvD5 doesn't suppress the differentiation and activation of DCs and doesn't polarize the M1 macrophages to M2 phenotype *in vitro*.
(A) Schematic representation of DC differentiation and experiment protocol. BM cells were isolated from 8-10-week-old SKG mice and culture for 5 days with GM-CSF and IL-4, with or without daily SPMs (RvD5: 1-100 nM). Cells were stained with anti-CD11b and anti-CD11c antibodies. **(B)** The frequency of CD11b＋CD11c＋cells were analyzed by flow cytometry. Data are representative of 5 independent experiments. **(C)** Schematic representation of DC activation and experiment protocol. On day5, cells were treated with or without RvD5 (1-1000 nM) and activated with or without LPS. One day after LPS activation, cells were stained with anti-CD11b, anti-CD11c, anti-CD86 and anti-MHC class Ⅱ antibodies. **(D)** The frequency of CD86+MHC class Ⅱ+ cells among CD11b+CD11c+ cells were analyzed by flow cytometry. Data are representative of 4 independent experiments. **(E)** Schematic representation of M1 macrophages and experiment protocol. BM cells were isolated and culture for 7 days with GM-CSF. On day 7, cells were treated with or without RvD5 (1-1000 nM) and with or without LPS/ IFNg to polarize to M1 phenotype. One day after LPS/ IFNg polarization, cells were stained with anti-CD11b, anti-F4/80, anti-CD86 and anti-CD206 antibodies. **(F)** The frequency of M1 phenotype (CD86+) and M2 phenotype (CD206+) cells among CD11b+F4/80+ cells were analyzed by flow cytometry. Data are representative of 4 independent experiments.

Supplemental figure 7

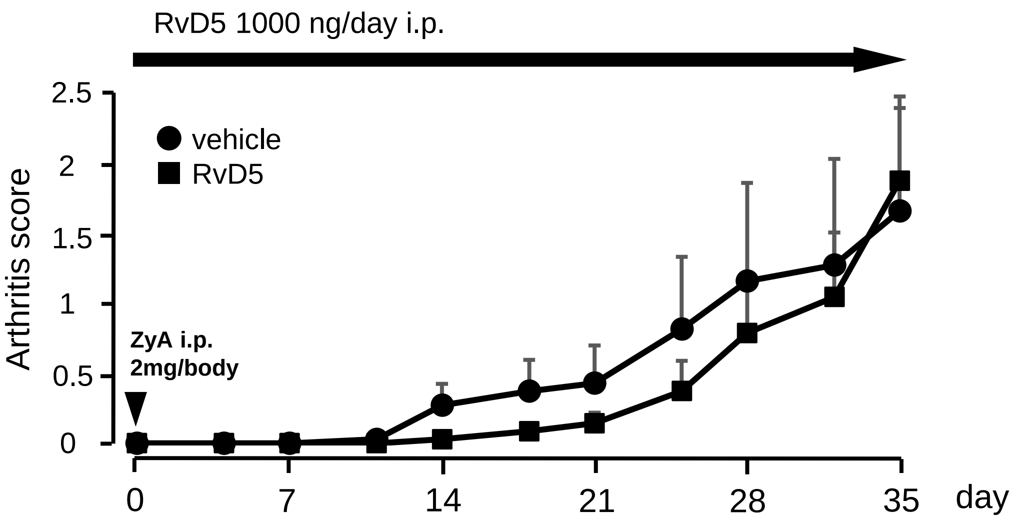

Supplemental figure 7. RvD5 treatment does not prevent arthritis in the SKG model *in vivo*.
SKG mice were induced arthritis with ZyA (2 mg/body i.p.) on day 0 and were given daily RvD5 (1000ng) i.p. from day 0 (RvD5, n = 7). Vehicle group were treated with NS (vehicle, n = 7). We recorded clinical arthritis score up to day 35.
